# Peer interaction and social responsibility among medical students: extracurricular activities as a mediator and achievement motivation as a moderator

**DOI:** 10.1080/10872981.2025.2585635

**Published:** 2025-11-19

**Authors:** Jing Tian, Zhidan Hui, Hongde Lei

**Affiliations:** a School of Education, Huazhong University of Science and Technology, Wuhan, Hubei Province, People's Republic of China

**Keywords:** Peer interaction, extracurricular activities, achievement motivation, social responsibility, medical students

## Abstract

Global healthcare challenges highlight the need to cultivate social responsibility in medical education. However, traditional teaching methods inadequately develop this competency, overlooking peer-driven mechanisms. This study proposes a moderated mediation model to explore how peer interaction impacts medical students' social responsibility, examining extracurricular activities as a mediator and achievement motivation as a moderator. Using cluster sampling, data from five waves (2014–2023) of the Student Survey of Learning and Development (SSLD) at a Chinese research university were analyzed, involving 6,546 medical students. Validated scales measured peer interaction, extracurricular activities, achievement motivation, and social responsibility. SPSS PROCESS with 5,000 bootstrap samples tested mediation and moderation effects. Peer interaction significantly and positively predicted medical students' social responsibility (*β* = 0.187, *p* < 0.001), with extracurricular activities serving as a partial mediator (indirect effect proportion: 66.51%). Crucially, achievement motivation moderated both the direct and the first half of the mediation path: Under low achievement motivation (*M−1SD*), the direct (*simple slope* = 0. 078, *t* = 3.723, *p* < 0. 001) and indirect (*simple slope* = 0. 387, *t* = 20.635, *p* < 0. 001) effects of peer interaction on social responsibility were statistically significant. Conversely, under high achievement motivation (*M + 1SD*), the direct effect became insignificant (*simple slope* = 0. 009, *t* = 0.546, *p* > 0. 05), and the indirect effect weakened (*simple slope* = 0. 295, *t* = 20.077, *p* < 0. 001). Peer interaction promotes social responsibility through extracurricular engagement, but high achievement motivation diminishes this effect. Students with strong extrinsic goals may misuse peer interaction and activities, undermining social responsibility. Structured peer support programs with reflective components are needed, especially for achievement-oriented students, to foster genuine social responsibility in medical education.

## Introduction

In the context of globalization and an aging population, the healthcare sector faces unprecedented challenges, such as the inequitable distribution of medical resources [1], strained physician‒patient relationships [2], and recurrent public health crises [3]. These challenges not only undermine healthcare accessibility and quality but also amplify the imperative for cultivating social responsibility among medical students—a competency critical for addressing evolving societal health demands. However, current pedagogical approaches demonstrate limited efficacy in fostering social responsibility [4]. Empirical evidence reveals troubling gaps: a multi-institutional survey of 1,083 students across 35 medical schools identified a significant disparity between medical students' self-assessed responsibility levels and the certification standards required for licensed physicians [5]. This deficit has tangible consequences, as students exhibiting underdeveloped social responsibility are at greater risk of diminished professional engagement, reduced learning motivation, and attrition from medical careers [6]. Conversely, students with robust social responsibility demonstrate greater capacity to sustain clinical enthusiasm, refine professional competencies, and align with the ethical imperatives of medical practice [7].

Efforts to increase social responsibility require systematic identification of its determinants. While social ecosystem theory emphasizes multifactorial influences, existing research disproportionately prioritizes curricular teaching over intrinsic behavioral mechanisms. For instance, Wren's quasi-experimental study of UAE institutions demonstrated that global citizenship curricula could improve social responsibility through structured reflective journals and field observations [8]. Nevertheless, two critical limitations persist. First, social responsibility development has pronounced temporal lag effects. Evaluations of Hong Kong's service-learning programs revealed delayed social responsibility improvements relative to other competencies despite optimized curricula [9], underscoring the complexity of its cultivation. Second, scholarly attention has overemphasized external pedagogical strategies while neglecting endogenous factors such as peer interaction dynamics—a notable oversight given medical education's reliance on collaborative learning. Moreover, existing research on peer interaction and social responsibility has focused primarily on scientists and engineers [10], with limited attention given to medical students. Medical students, who engage in extensive theoretical and clinical learning, exhibit a strong need for peer interaction, not only for academic exchange [11] but also for emotional support and mutual understanding [12]. Crucially, neither the mechanisms linking peer interaction to social responsibility nor evidence-based strategies for leveraging these relationships have been established, hindering the translation of student-centered educational principles into practice.

To address the above research gaps, this study integrates social support theory, social cognitive theory, and self-determination theory to investigate (1) the relationship between peer interaction and social responsibility among medical students; (2) the mediating role of extracurricular activities; and (3) the moderating effect of achievement motivation. By elucidating *how* peer interaction influences social responsibility and *under what conditions* its effects are more pronounced, this study aims to deepen the understanding of the mechanisms underlying the relationship between peer interaction and social responsibility and to offer insights for cultivating ethical, socially responsible medical professionals through a peer-driven educational framework.

### The relationship between peer interaction and social responsibility

Peer interaction is defined as the reciprocal exchange of support and active participation among individuals of equal status to facilitate knowledge acquisition and skill development [13]. This collaborative dynamic fosters resource sharing, mutual encouragement, and joint engagement in learning environments [14]. Previous research has demonstrated the positive effects of peer interaction on adolescent development and mental health [15], suggesting its significant influence on individual cognition and behavior [16], including the enhancement of social responsibility. Empirical studies have consistently reported a positive correlation between peer interaction and social responsibility [17]. For example, a longitudinal study of 128 university students (2017–2019, measured via the Generalized Professional Responsibility Assessment scale) revealed that peer interaction, as a form of community engagement, significantly predicts the development of social responsibility, particularly among non-white student populations [18]. Another mixed-methods study (Seas Suas program) involving 193 university students demonstrated that structured peer support interventions led to significant improvements in empathy, social responsibility, and prosocial confidence while effectively counteracting the inhibitory effects of the bystander phenomenon on responsibility adoption [19].

Building upon these empirical findings, social support theory further explains that peer interaction, as a key source of social support, facilitates the development of social responsibility through three mechanisms: emotional support, informational support, and instrumental support [20]. More specifically, this manifests through (a) *emotional support* whereby peer groups foster professional identity formation through empathy, identification, and role modeling [16]; (b) *informational support* where case-based discussions and critical incident debriefings enhance individuals' awareness and critical thinking about social issues [21]; and (c) *instrumental support* through knowledge sharing and perspective exchange provide practical avenues for fulfilling social responsibility [22]. Although these relationships are well established in general student populations, their applicability to medical students remains underexplored, particularly given the distinct clinical training pressures and professional socialization challenges unique to medical education. Therefore, this study proposes Hypothesis 1: Peer interaction positively predicts medical students' social responsibility.

### The mediating role of extracurricular activities

Extracurricular activities serve as an effective platform for cultivating well-rounded individuals by providing opportunities to develop talents, enhancing physical and mental well-being, and facilitating social transition after graduation [23]. Empirical evidence consistently underscores the positive association between extracurricular participation and social responsibility. For example, a longitudinal study involving 539 adolescents revealed that school-based extracurricular opportunities significantly predict adolescents' engagement in community service and prosocial behaviors [24]. Furthermore, active participation in extracurricular activities allows students to recognize their societal value, thereby motivating them to assume social responsibility. An educational intervention study among adolescents revealed that participation in socio-cultural programs (a form of extracurricular activity) significantly enhanced social responsibility, as evidenced by improvements in empathy, awareness of social norms, and responsible decision-making [25].

The link between peer interaction and extracurricular participation is well documented in educational research. Schaefer et al. (2023) conducted a large-scale survey using stochastic actor-oriented modeling for two-mode network analysis, providing empirical evidence that both friendship ties and comembership relations significantly influence adolescents' extracurricular activity participation patterns [26]. Experimental research by Pong and Leung demonstrated that Hong Kong business students who participated in community service learning showed greater improvements in career adaptability, ethical perspectives, and social responsibility than nonparticipants did [27]. Grounded in social cognitive theory [28], extracurricular activities function as transformational mediators between peer interaction and social responsibility. Notably, while extracurricular activities are frequently examined as independent predictors in medical education research [29], their mediating role between peer interaction and social responsibility remains underexplored—particularly within the unique context of medical education. Therefore, this study proposes Hypothesis 2: Peer interaction indirectly influences medical students' social responsibility through extracurricular activities.

### Moderating role of achievement motivation

According to self-determination theory [30], achievement motivation represents a multidimensional construct encompassing both intrinsic (e.g., personal growth) and extrinsic (e.g., academic performance, career advancement) dimensions [31]. This study specifically examines extrinsic motivation components, including academic performance, graduate school preparation, and career development [32]. As a dynamic psychological trait, achievement motivation exhibits marked plasticity in modulating cognitive-behavioral outcomes [33], thereby serving as an important moderator in understanding how various factors influence cognition and behavior [34].

The moderating effects of achievement motivation manifest through two primary mechanisms: cognitive processing and behavioral regulation. From a cognitive perspective, achievement motivation influences the relationship between peer interaction and social responsibility by modulating information processing patterns. Social cognitive theory suggests that individuals with high achievement motivation tend to adopt goal-directed attention strategies [35], preferentially selecting information directly related to academic achievement during peer interactions (e.g., exam preparation, research opportunities) while neglecting value-based content concerning social responsibility (e.g., cultural identity, ethical norms) [36]. However, empirical studies examining the moderating effect of achievement motivation on the peer interaction-social responsibility relationship remain limited.

From a behavioral perspective, achievement motivation operates through the resource competition model. Self-determination theory's motivational continuum [37] suggests that achievement motivation regulates the type of behavioral drive underlying extracurricular participation. Students with high achievement motivation tend to rely on external regulation [38] (e.g., pursuing grades, career competitiveness), potentially transforming extracurricular activities into resume-building tools [39]. Research indicates that individuals with high levels of achievement motivation exhibit performance patterns that are highly dependent on achievement incentives. For example, Julia Schüler and Wanja Wolff's (2020) experimental study revealed that when tasks lack clear achievement incentives, high-achievement-motivation individuals perform worse than their low-motivation counterparts do [40]. This suggests that individuals with high levels of achievement motivation may prioritize instrumental goals (e.g., grades, career advancement) over intrinsic values (e.g., social responsibility, ethical norms).

These moderating mechanisms appear particularly pronounced in medical education contexts. The prevailing meritocracy culture in medical education systems encourages medical students with high achievement motivation to approach both peer interaction and extracurricular activities as resource competition strategies [41]. Such instrumental learning behaviors and interpersonal patterns may hinder medical students' social responsibility development. On the basis of these observations, this study proposes Hypothesis 3: Achievement motivation moderates both the direct relationship between peer interaction and medical students' social responsibility and the mediating pathway through extracurricular activities. Specifically, both the direct effect of peer interaction on social responsibility and the mediating effect of extracurricular activities are subject to moderation by achievement motivation.

**Figure 1.**
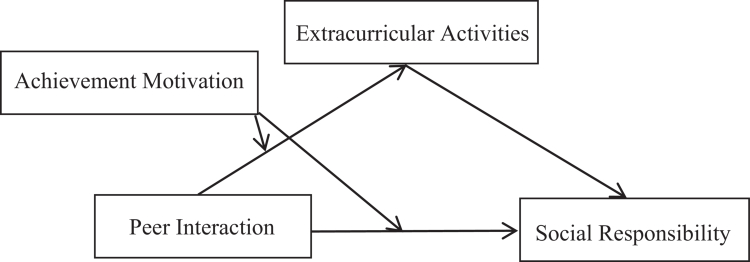
Hypothetical model.

To sum up, by integrating social support theory, social cognitive theory, and self-determination theory, this study establishes a moderated mediation model (Figure 1) to examine the relationships among peer interaction, extracurricular activities, achievement motivation, and medical students' social responsibility. The findings offer both empirical evidence and theoretical insights for enhancing medical students' social responsibility development.

## Methods

### Participants

This study employed a cluster sampling approach to select participants from five waves (2014, 2016, 2018, 2021, and 2023) of the SSLD administered at a research-intensive Chinese university. The final analytical sample comprised 6,546 validated responses from medical students, stratified as follows: 1,504 (23.0%) first-year students, 1,569 (24.0%) second-year students, 1,559 (23.8%) third-year students, 1,099 (16.8%) fourth-year students, and 815 (12.5%) fifth-year students.

### Measures and instruments

The study employed the SSLD questionnaire, a comprehensive self-report measure originally developed by a research-intensive Chinese university, as its primary measurement instrument. This tool assesses five key domains—academic expectancy, school support, assessment and feedback, student engagement, and learning outcomes—through 21 distinct indicators [42]. Previous psychometric evaluations have demonstrated the robust reliability and validity of the instrument. Initial administration in May 2014 achieved a response rate of 37.66% (12,134/32,220 eligible participants), with 93.09% of the returned questionnaires meeting validity criteria (11,295 valid responses). Reliability analyzes revealed strong internal consistency across all the SSLD indicators, with Cronbach's *α* coefficients distributed as follows: five scales between 0.7–0.8, twelve between 0.8–0.9, and four exceeding 0.9 [43]. Further validation through exploratory factor analysis confirmed the instrument's structural validity, whereas correlation analyzes supported its criterion-related validity, collectively satisfying established measurement standards.

The current analysis focused on four specific indicators: peer interaction (Cronbach's *α* = 0.612), extracurricular activities (Cronbach's *α* = 0.719), achievement motivation (Cronbach's *α* = 0.695), and social responsibility (Cronbach's *α* = 0.869). These variables were examined across five medical student cohorts (2014, 2016, 2018, 2021, and 2023). Exploratory factor analysis of the combined data set produced an overall Cronbach's *α* coefficient of 0.815 for these measures across all survey waves, demonstrating excellent scale reliability. The details of each variable in this study are outlined as follows:

### Peer interaction

The peer interaction scale consists of six items aimed at assessing the frequency of interactions among students within various peer groups, including roommates, classmates, and club members. The responses were rated on a four-point Likert scale ranging from 1 (‘Never’) to 4 (‘Very often’). A higher score reflects a greater degree of peer interaction. Exploratory factor analysis yielded a Kaiser‒Meyer‒Olkin (KMO) measure of 0.687, and Bartlett's test of sphericity was significant (*χ²* = 7611.178, *df* = 15, *p* < 0.001), confirming the data's suitability for factor analysis. All item loadings ranged from 0.576−0.823. Confirmatory factor analysis (CFA) demonstrated good model fit: the goodness-of-fit index (GFI) was 0.999, the adjusted goodness-of-fit index (AGFI) was 0.988, the comparative fit index (CFI) was 0.999, the Tucker‒Lewis index (TLI) was 0.979, and the root mean square error of approximation (RMSEA) was 0.040, indicating satisfactory construct validity. In this study, the scale's internal consistency was acceptable, with a Cronbach's *α* coefficient of 0.612.

### Extracurricular activities

The extracurricular activities scale consists of five items aimed at assessing diverse aspects, such as participating in club activities, joining social practices, and taking part in campus cultural events (example item: ‘I regularly participate in volunteer activities’). The responses were rated on a four-point Likert scale ranging from 1 (‘Never’) to 4 (‘Very often’). A higher score indicates greater involvement in extracurricular activities. Exploratory factor analysis revealed a KMO measure of sampling adequacy of 0.703, and Bartlett's test of sphericity was significant (χ² = 6954.283, *df* = 10, *p* < 0.001), confirming the data's suitability for factor analysis. All item loadings ranged from 0.537−0.777. The CFA demonstrated good model fit: the GFI was 0.998, the AGFI was 0.971, the CFI was 0.996, the TLI was 0.956, and the RMSEA was 0.069, indicating satisfactory construct validity. In this study, the scale's internal consistency was acceptable, with a Cronbach's *α* coefficient of 0.719.

### Achievement motivation

The achievement motivation scale consists of six items aimed at assessing multidimensional goal-setting behaviors related to academic and professional aspirations, such as achieving high academic performance, preparing for graduate study domestically or abroad, enhancing employability skills, seeking high-paying jobs after graduation, and preparing for entrepreneurship (example item: ‘I should acquire the skills needed for future employment as much as possible’). Responses were assessed via a four-point Likert scale ranging from 1 (‘Not important’) to 4 (‘Very important’). Exploratory factor analysis demonstrated adequate sampling suitability, with a KMO measure of 0.739. Bartlett's test of sphericity was significant (*χ²* = 6603.349, *df* = 15, *p* < 0.001), confirming the data's suitability for factor analysis. All item loadings ranged from 0.537−0.727. The CFA demonstrated good model fit: the GFI was 0.998, the AGFI was 0.988, the CFI was 0.994, the TLI was 0.977, and the RMSEA was 0.039, indicating satisfactory construct validity. In this study, the scale's internal consistency was acceptable, with a Cronbach's *α* coefficient of 0.695.

### Social responsibility

The social responsibility scale consists of six items aimed at assessing diverse aspects, such as the understanding of personal social responsibility, adherence to basic social and professional ethical norms, identification with the national cultural spirit, understanding and tolerance of international cultural diversity, and personal moral cultivation (example item: ‘I have a good understanding of basic social and professional ethical norms and always follow them’). The responses were rated on a four-point Likert scale ranging from 1 (‘No improvement’) to 4 (‘Great improvement’). Exploratory factor analysis revealed excellent sampling adequacy, with a KMO measure of 0.860. Bartlett's test of sphericity was significant (*χ²* = 16510.274, *df* = 10, *p* < 0.001), confirming the data's suitability for factor analysis. All item loadings ranged from 0.709−0.875. The CFA demonstrated good model fit: the GFI was 0.997, the AGFI was 0.987, the CFI was 0.997, the TLI was 0.992, and the RMSEA was 0.044, indicating satisfactory construct validity. In this study, the scale's internal consistency was acceptable, with a Cronbach's *α* coefficient of 0.869.

### Statistical analysis

The data analysis was conducted via SPSS 26.0 and AMOS 26.0. Initially, Harman's single-factor test was performed to examine common method bias. Descriptive statistics and correlation analyzes were employed to assess the relationships among four key variables: peer interaction, extracurricular activities, achievement motivation, and social responsibility. Subsequent analyzes utilized the PROCESS macro for mediation and moderation analyzes [44]. Model 4 was applied to examine the mediating role of extracurricular activities in the relationship between peer interaction and medical students' social responsibility. Model 8 was implemented to test (1) the moderating effect of achievement motivation on the direct relationship between peer interaction and social responsibility and (2) the moderating effect of achievement motivation on the first stage of the mediation pathway (peer interaction → extracurricular activities → social responsibility). All the mediation and moderation effects were verified via nonparametric bootstrapping with 5,000 resamples to generate bias-corrected confidence intervals.

## Ethical approval

This study received ethical approval from the Psychological Ethics Committee of Huazhong University of Science and Technology (Approval No. 202305) and was conducted in accordance with the Declaration of Helsinki. The study utilized an online survey system developed by the university. All participants provided informed consent and completed the questionnaire voluntarily and anonymously. The collected data were treated with strict confidentiality and were accessible only to authorized members of the research team for academic purposes.

## Results

### Common method bias test

This study utilized Harma's single-factor test to assess common method bias. The test resulted in 6 eigenvalues exceeding 1, with the primary factor explaining 22.398% of the total variance. This figure, which falls short of the 40% benchmark, suggests that common method bias does not significantly affect the research findings [45].

### Descriptive statistics correlations between the study variables

Correlation analysis revealed significant positive associations between social responsibility and peer interaction (*r* = 0.205, *p* < 0.01), extracurricular activities (*r* = 0.345, *p* < 0.01), and achievement motivation (*r* = 0.329, *p* < 0.01), as presented in Table 1.

**Table 1.** Pearson correlation analysis among different variables.

Variables	M ± SD	1	2	3	4
1.Peer interaction	2.440 ± 0.577	1			
2.Extracurricular activities	2.732 ± 0.602	0.327**	1		
3.Achievement motivation	3.060 ± 0.498	0.251**	0.259**	1	
4.Social responsibility	3.009 ± 0.660	0.205**	0.345**	0.329**	1

Note: *N* = 6546. *p < 0.05, **p < 0.01, ***p < 0.001 (two-tailed tests).

**Table 2.** Mediation model test of extracurricular activities.

Regression equation		Model fit indices			Coefficient significance	
Outcome variable	Predictor variable	*R*	*R* ^*2*^	*F*	*β*	*t*
Social responsibility		0.243	0.059	102.208***		
	Gender				0.048	3.968***
	Grade				0.038	3.094**
	Year of survey				0.107	8.625***
	Peer interaction				0.187	15.209***
Extracurricular activities		0.370	0.137	259.864***		
	Gender				0.014	1.245
	Grade				−0.051	−4.348***
	Year of survey				−0.163	−13.744***
	Peer interaction				0.358	30.518***
Social responsibility		0.402	0.162	252.618***		
	Gender				0.043	3.767***
	Grade				0.055	4.796***
	Year of survey				0.163	13.761***
	Extracurricular activities				0.346	28.356***
	Peer interaction				0.063	5.066***

Note: N = 6546. *p < 0.05, **p < 0.01, ***p < 0.001 (two-tailed tests).

### Peer interaction and social responsibility among medical students: testing the mediating role of extracurricular activities

The independent samples t test revealed statistically significant gender differences in peer interaction (*t* = −2.890, *p* < 0.01) and social responsibility (*t* = 3.914, *p* < 0.001). The one-way ANOVA results further revealed significant grade variations in peer interaction (*F* = 5.127, *p* < 0.001), extracurricular activities (*F* = 13.884, *p* < 0.001), achievement motivation (*F* = 5.845, *p* < 0.001), and social responsibility (*F* = 4.518, *p* < 0.01). Similarly, significant disparities were observed across years of survey for peer interaction (*F* = 278.682, *p* < 0.001), extracurricular activities (*F* = 28.191, *p* < 0.001), achievement motivation (*F* = 80.504, *p* < 0.001), and social responsibility (*F* = 40.986, *p* < 0.001). Consequently, gender, grade, and year of survey were included as control variables in subsequent analyzes.

Using Model 4 of Hayes' (2012) PROCESS macro for SPSS to test the mediating effect in the relationship between peer interaction and social responsibility while controlling for gender, grade, and year of survey, the results (Tables 2 and 3) demonstrated that peer interaction significantly predicted social responsibility (*β* = 0.187, *t* = 15.209, *p* < 0.001). When the mediator (extracurricular activities) was included in the model, the direct effect of peer interaction on social responsibility remained significant (*β* = 0.063, *t* = 5.066, *p* < 0.001). Peer interaction also significantly predicted extracurricular activities (*β* = 0.358, *t* = 30.518, *p* < 0.001), which in turn significantly predicted social responsibility (*β* = 0.346, *t* = 28.356, *p* < 0.001). Bootstrap analysis with 95% confidence intervals revealed that neither the direct effect nor the indirect effect through extracurricular activities included zero in their intervals (Table 3), indicating that peer interaction not only directly predicts social responsibility but also exerts an indirect effect through extracurricular activities. The direct effect (0.072) accounted for 33.49% of the total effect (0.215), whereas the indirect effect (0.143) accounted for 66.51%.

**Table 3.** Decomposition of total, direct, and indirect effects​.

	Effect	Boot SE	Boot LLCI	Boot ULCI	Effect size
Total effect	0.215	0.014	0.188	0.243	
Direct effect	0.072	0.014	0.044	0.100	33.49%
Indirect effect	0.143	0.007	0.129	0.158	66.51%

### Peer interaction and social responsibility among medical students: testing a moderated mediation model

Hayes' (2012) PROCESS macro (Model 8) was employed to examine the moderated mediation model, controlling for gender, grade, and year of survey. Model 8 was selected because it aligns with the theoretical framework of this study, testing moderation effects on both the direct path (peer interaction → social responsibility) and the first stage of the mediation pathway (peer interaction → extracurricular activities). The results (Table 4) demonstrated that after incorporating achievement motivation into the model, the interaction term (peer interaction × achievement motivation) had significant negative predictive effects on both social responsibility (*β* = −0.069, *t* = −2.900, *p* < 0.01) and extracurricular activities (*β* = −0.092, *t* = −4.187, *p* < 0.001). These findings indicate that achievement motivation not only moderates the direct relationship between peer interaction and social responsibility but also significantly influences the association between peer interaction and extracurricular activities.

**Table 4.** Results of the moderated mediation model analysis.

Regression equation		Model fit indices			Coefficient significance	
Outcome variable	Predictor variable	*R*	*R* ^*2*^	*F*	*β*	*t*
Extracurricular activities		0.423	0.179	237.185***		
	Gender				0.018	1.308
	Grade				−0.017	−3.202**
	Year of survey				−0.037	−15.973***
	Peer interaction				0.623	8.699***
	Achievement motivation				0.480	8.463***
	PI × AM				−0.092	−4.187***
Social responsibility		0.458	0.210	247.700***		
	Gender				0.059	4.034***
	Grade				0.034	6.072***
	Year of survey				0.028	11.071***
	Extracurricular activities				0.321	24.137***
	Peer interaction				0.254	3.276**
	Achievement motivation				0.478	7.785***
	PI × AM				−0.069	−2.900**

Note: *N* = 6546; *p < 0.05, **p < 0.01, ***p < 0.001 (two-tailed tests); PI×AM, Peer Interaction × Achievement Motivation.

**Figure 2.**
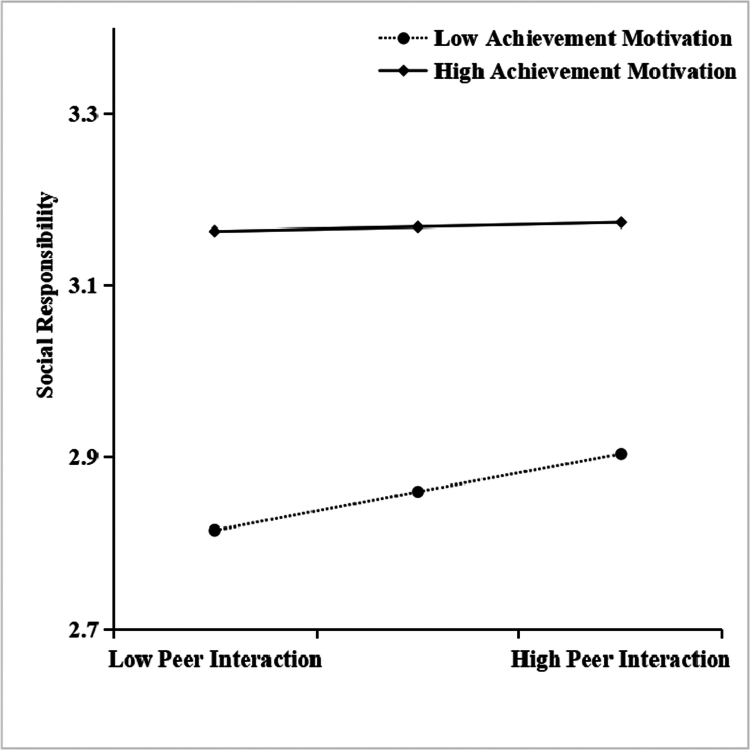
Moderating role of achievement motivation in the relationship between peer interaction and social responsibility.

**Figure 3.**
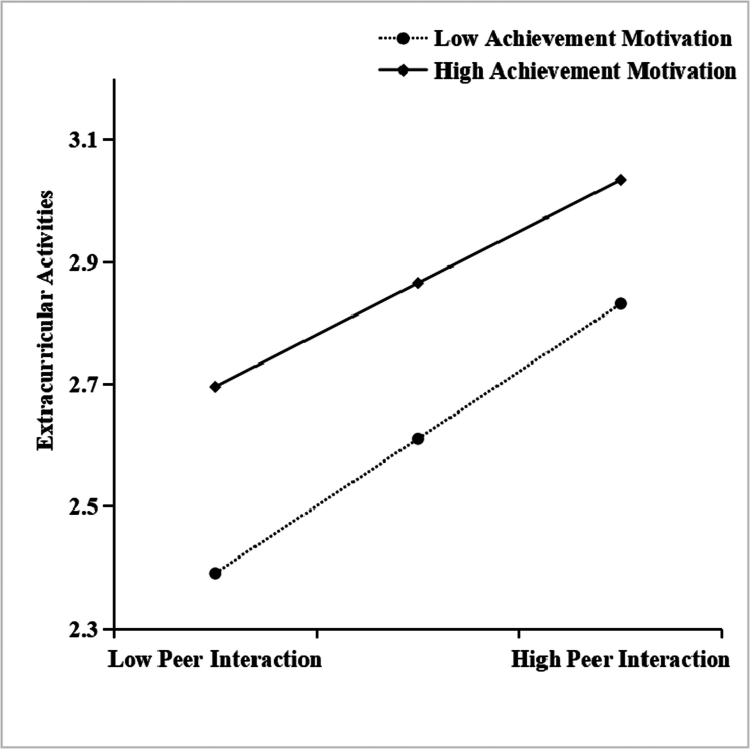
Moderating role of achievement motivation in the relationship between peer interaction and extracurricular activities.

The results of simple slope analysis (Figures 2, 3) revealed significant patterns in the moderating effects. As shown in Figure 2, when achievement motivation was low (*M-1SD* = 2.562), peer interaction had a significant positive effect on social responsibility (*simple slope* = 0. 078, *t* = 3.723, *p* < 0. 001). At the mean level (*M* = 3.060), the effect decreased in magnitude but remained significant (*simple slope* = 0.043, *t* = 2.986, *p* < 0. 001). Notably, at high levels (*M + 1SD* = 3.558), the effect was no longer significant (*simple slope* = 0. 009, *t* = 0.546, *p* > 0. 05), suggesting a dose-dependent attenuation pattern.

Figure 3 shows that the mediation effect size was significantly greater in the low-achievement motivation group (*simple slope* = 0. 387, *t* = 20.635, *p* < 0. 001) compared with both the mean (*simple slope* = 0. 341, *t* = 26.695, *p* < 0. 001) and high-achievement motivation groups (*simple slope* = 0. 295, *t* = 20.077, *p* < 0. 001), showing a systematic decreasing trend. These results suggest that the indirect effect of peer interaction on social responsibility through extracurricular activities becomes progressively weaker as achievement motivation levels increase.

## Discussion

By integrating social support theory, social cognitive theory, and self-determination theory, this study establishes a moderated mediation model to examine how peer interaction influences medical students' social responsibility, with extracurricular activities as a mediator and achievement motivation as a moderator.

### The mediating role of extracurricular activities

This study revealed that peer interaction positively predicts medical students' social responsibility through the mediating role of extracurricular activities (supporting H1 and H2). This finding aligns with social support theory and social cognitive theory [20,28].

On the one hand, the pathway through which peer interaction influences social responsibility via extracurricular activities can be systematically explained by the three-dimensional framework of social support theory. The theory categorizes social support into emotional support, informational support, and instrumental support, which collectively elucidate the mechanisms by which peer interaction promotes participation in extracurricular activities. First, peer interaction lowers the psychological barriers to extracurricular engagement by providing a sense of acceptance and belonging (emotional support) [12]. For example, the ‘Big Sibling Mentoring Program’ implemented at King Saud University College of Medicine demonstrates the critical role of emotional support. This mentoring initiative pairs senior students with first-year medical students, significantly enhancing the latter's motivation to participate in extracurricular activities [46]. Second, peer interaction improves the efficacy of extracurricular participation through knowledge sharing (informational support) [47]. A cross-sectional study at Busitema University School of Medicine in Uganda, which employed semistructured questionnaires and Bloom's taxonomy to assess medical students' involvement in an antimicrobial resistance club, highlighted the importance of informational support in extracurricular engagement [48]. Third, peer interaction removes practical obstacles to extracurricular participation by offering tangible assistance (instrumental support) [49]. For example, the ‘USMLE Study Group’ project at Tokushima University School of Medicine in Japan used a quasiexperimental design to demonstrate how near-peer teaching facilitated medical students' participation in extracurricular activities such as international medical English learning [50].

On the other hand, the mediating role of extracurricular activities in the relationship between peer interaction and social responsibility is supported by the triadical reciprocal determinism model of social cognitive theory [28]. At the behavioral level, extracurricular activities serve as a practical arena for medical students to translate social support from peer interactions (e.g., emotional resonance, knowledge sharing) into concrete social responsibility actions. For example, Fujii et al. employed mixed methods to systematically investigate students across all five years of medical school and reported that extracurricular activities functioned as a transformative space where social support was converted into responsible behaviors [51]. At the cognitive level, extracurricular activities increase the impact of peer interaction on social responsibility through reflective practices. A study integrating structured extracurricular activities—such as memorial ceremonies for ‘silent mentors,’ clinical ethics group discussions, and medical humanities competitions—demonstrated that reflective practices significantly strengthened medical students' sense of social responsibility [52]. In summary, peer interaction enhances medical students' participation in extracurricular activities, thereby fostering their social responsibility.

### The moderating role of achievement motivation

This study constructs a moderated mediation model to examine the role of achievement motivation in the relationships among peer interaction, extracurricular activities, and social responsibility. The results indicate that achievement motivation moderates not only the direct relationship between peer interaction and social responsibility but also the first half of the mediation pathway (peer interaction → extracurricular activities → social responsibility) (supporting H3). This finding provides a novel theoretical perspective on the dynamic interplay between individual motivation and social responsibility development in medical education.

Specifically, the direct predictive effect of peer interaction on social responsibility is more pronounced among medical students with low levels of achievement motivation than among their high-achieving counterparts. This result diverges from prior research. For example, Nshimiyimana and Cartledge reported that peer-assisted learning enhances medical students' intrinsic motivation, thereby promoting skill development [53]. This discrepancy may stem from two factors: methodological differences in survey instruments and variations in the conceptualization of motivation. In this study, achievement motivation primarily reflects extrinsic motivation, such as pursuing grades, postgraduate education, and career advancement [32]. Extrinsic motivation tends to intensify as medical students progress through their training [54], influencing their self-efficacy and academic satisfaction [55]. However, students with high levels of achievement motivation may focus excessively on externally defined standards, leading to social comparisons and negative self-evaluations [56], which can diminish their sense of social responsibility. The unique context of medical education amplifies this moderating effect. The ‘knowledge-intensive’ nature of medical training predisposes students with high achievement motivation to narrow peer interactions into ‘exam-focused collaborations’ (e.g., case discussions, knowledge reviews) [57] rather than broader dialogs on social responsibility. This selective information processing may result in the neglect of socially relevant issues, weakening the positive impact of peer interaction.

Furthermore, this study reveals that peer interaction more effectively promotes extracurricular participation and social responsibility among students with low levels of achievement motivation. This finding aligns with the motivational continuum model of self-determination theory [37]. The measured achievement motivation in this study primarily exhibited characteristics of external regulation and introjected regulation—behaviors driven by external rewards (e.g., grades, career advancement) or internal pressures (e.g., avoiding failure) rather than genuine value internalization [39]. For example, medical students with high achievement motivation often instrumentalize peer interactions and extracurricular activities, viewing them as steppingstones for career advancement. A Brazilian study of 221 medical students revealed that their primary motivation for participating in extracurricular activities was to enhance their resumes and develop professional skills [58]. In contrast, students with low achievement motivation, who are less constrained by external goals, benefit more from peer interaction through emotional [46], informational [48], and instrumental support [50], which fosters extracurricular participation and internalizes social responsibility. Thus, peer interaction more readily influences the engagement of medical students with low achievement motivation in extracurricular activities, thereby enhancing their social responsibility.

### Implications for medical education

This study constructs a moderated mediation model to elucidate the mechanism and boundary conditions through which peer interaction influences medical students' social responsibility, providing some theoretical contributions and practical implications for fostering social responsibility in medical education. In terms of theoretical contributions, first, this research integrates social support theory, social cognitive theory, and self-determination theory to explain the intrinsic mechanism by which peer interaction enhances social responsibility through the mediating role of extracurricular activities. Second, the study reveals the moderating effect of achievement motivation, demonstrating that high achievement motivation may attenuate the positive impact of peer interaction on social responsibility. This finding extends the theoretical understanding of the relationship between motivation and social responsibility in medical education, offering new insights into individual differences in medical students' social responsibility development.

With respect to practical implications, medical schools should recognize the unique role of peer interaction in cultivating social responsibility. Structured peer support programs (e.g., mentorship initiatives and peer learning groups) are expected to encourage participation in extracurricular activities [59], with particular attention given to medical students with high levels of achievement motivation. Simultaneously, strategies for guiding achievement motivation should be optimized to avoid excessive emphasis on utilitarian goals. For example, integrating reflective practices on social responsibility issues into extracurricular activities can help high-achievement-motivation students balance professional development and social value internalization. Furthermore, educational administrators should design integrated intervention programs that include peer interaction, extracurricular activities, and a formal curriculum to enhance the systematic and sustainable cultivation of social responsibility.

### Limitations and prospects

Although this study revealed the mechanism through which peer interaction influences medical students' social responsibility via extracurricular activities, as well as the moderating role of achievement motivation, three main limitations should be acknowledged. First, the cross-sectional design makes it difficult to establish causal relationships between variables. Future research could employ longitudinal tracking or experimental designs to further verify the temporal sequence of the mediating and moderating effects. Second, the sample was drawn from only one research-intensive university in China, and no distinction was made between clinical and nonclinical specialties, which may limit the generalizability of the findings. Subsequent studies could expand sample diversity through cross-cultural comparisons and multi-institutional sampling. Third, the measurement of achievement motivation focused primarily on extrinsic dimensions (e.g., academic performance, career development), while insufficient attention was given to examining the moderating effects of intrinsic motivation (e.g., interest, altruism). Future research could adopt a more comprehensive assessment by integrating the motivational continuum from self-determination theory.

## Conclusions

The findings reveal that extracurricular activities partially mediate the positive effect of peer interaction on social responsibility. Meanwhile, achievement motivation moderates both the direct path (peer interaction → social responsibility) and the first stage of the mediation pathway (peer interaction → extracurricular activities). These findings contribute to the theoretical understanding of the role of peer interaction in shaping social cognition and prosocial behaviors in medical education. Furthermore, they offer practical insights for developing peer-based interventions that incorporate individual differences in achievement motivation.

## Data Availability

There is a data set associated with this submission. The data set is deposited in Harvard Dataverse, and its location is https://doi.org/10.7910/DVN/UPBGT4.

## References

[1] Chen S, Lai K-H, Guo X, et al. The influence of digital health technology on the allocation of regional medical resources in China. Health Policy Technol. 2025;14(3):1, 101013. doi: 10.1016/j.hlpt.2025.101013

[2] Han Y, Lie RK, Li Z, et al. Trust in the doctor‒patient relationship in Chinese public hospitals: evidence for hope. Patient Prefer Adherence. 2022;16:647–657. doi: 10.2147/PPA.S352636 35283627 PMC8910463

[3] Huang S-W, Liou JJH, Chuang H-H, et al. Exploring the key factors for preventing public health crises under incomplete information. Int J Fuzzy Syst. 2021;23(8):2467–2488. doi: 10.1007/s40815-021-01157-z

[4] Demirören M, Atılgan B. Impacts of service learning-based social responsibility training on medical students. Adv Physiol Educ. 2023;47(2):166–174. doi: 10.1152/advan.00049.2022 36701494

[5] Bußenius L, Harendza S, van den Bussche H, et al. Final-year medical students' self-assessment of facets of competence for beginning residents. BMC Med Educ. 2022;22(1):82. doi: 10.1186/s12909-021-03039-2 35130891 PMC8822672

[6] Gerull KM, Pérez M, Cipriano CA, et al. Evolution of medical students' interest in orthopedic surgery careers from matriculation to graduation. JBJS Open Access. 2024;9(3):e24.00019. doi: 10.2106/JBJS.OA.24.00019 PMC11216667 38957705

[7] Özdinç A, Aydin Z, Salman Ö. A positive correlation between idealist ethics and psychological well-being: a cross-sectional study among future physicians. Bangladesh J Med Sci. 2025;24(1):164–176. doi: 10.3329/BJMS.V24I1.78730

[8] Wren S. Improving Emirati students' social responsibility competence through global citizenship education. Learn Teach High Educ Gulf Perspect. 2021;17:80–94. doi: 10.1108/LTHE-09-2020-0046

[9] Chan SCF, Grace N, Ho-Yin YJ, et al. Enhancing the impacts of international service-learning on intercultural effectiveness and global citizenship development through action research. Educ Action Res. 2022;30(3):526–541. doi: 10.1080/09650792.2020.1860106

[10] Alimin M, Mun J, Lee H. Investigating perceptions of the social responsibility of scientists and engineers: comparison among South Korean, Malaysian, and Indonesian university students in STEM fields. Asia Pac Sci Educ. 2024;10:1–31. doi: 10.1163/23641177-bja10088

[11] Souza AD, Punja D, Prabhath S, et al. Influence of pretesting and a near peer sharing real life experiences on CPR training outcomes in first year medical students: a nonrandomized quasiexperimental study. BMC Med Educ. 2022;22(1):434. doi: 10.1186/s12909-022-03506-4 35668395 PMC9172151

[12] Puranitee P, Kaewpila W, Heeneman S, et al. Promoting a sense of belonging, engagement, and collegiality to reduce burnout: a mixed methods study among undergraduate medical students in a non-Western, Asian context. BMC Med Educ. 2022;22(1):327. doi: 10.1186/s12909-022-03380-0 35484548 PMC9047274

[13] Topping K, Buchs C, Duran D, et al. Effective peer learning: from principles to practical implementation. London: Routledge. 2017.

[14] Li R, Lin X. Factors influencing peer interaction among college students in blended learning environments: a study based on SEM and ANN. Interact Learn Environ. 2024;33:1–25. doi: 10.1080/10494820.2024.2440880

[15] Orben A, Tomova L, Blakemore SJ. The effects of social deprivation on adolescent development and mental health. Lancet Child Adolesc Health. 2020;4(8):634–640. doi: 10.1016/S2352-4642(20)30186-3 32540024 PMC7292584

[16] Pan F, Zhu G, Sui W, et al. The effects of peer interaction on learning outcome of college students in digital environment: the chain-mediated role of attitude and self-efficacy. Stud Educ Eval. 2024;83:101404. doi: 10.1016/j.stueduc.2024.101404

[17] Schiff D, Logevall E, Borenstein J, et al. Linking personal and professional social responsibility development to microethics and macroethics: observations from early undergraduate education. J Eng Educ. 2021;110(1):70–91. doi: 10.1002/jee.20371

[18] Schiff DS, Lee J, Borenstein J, et al. The impact of community engagement on undergraduate social responsibility attitudes. Stud High Educ. 2024;49(7):1151–1167. doi: 10.1080/03075079.2023.2260414

[19] Brady B, Sylvester C, Hughes B, et al. Stand up/Seas Suas: promoting peer awareness, empathy and helping among third level students. Pastor Care Educ. 2023;41(2):225–244. doi: 10.1080/02643944.2022.2054022

[20] House JS, Umberson D, Landis KR. Structures and processes of social support. Annu Rev Sociol. 1988;14:293–318. doi: 10.1146/annurev.so.14.080188.001453

[21] Geng X, Zhan Y, You H, et al. Exploring the characteristics of undergraduates' critical thinking development in peer interaction via epistemic network analysis. Think Skills Creat. 2024;52:101553. doi: 10.1016/j.tsc.2024.101553

[22] Su JJ, Paguio J, Baratedi WM, et al. Experience of coronary heart disease patients with a nurse-led eHealth cardiac rehabilitation: qualitative process evaluation of a randomized controlled trial. Heart Lung. 2023;57:214–221. doi: 10.1016/j.hrtlng.2022.10.005 36265371

[23] Rubin RS, Bommer WH, Baldwin TT. Using extracurricular activity as an indicator of interpersonal skill: prudent evaluation or recruiting malpractice?. Hum Resour Manag J. 2002;41(4):441–454. doi: 10.1002/hrm.10053

[24] Le T, Johnson S, Lerner J. The apple does not fall far from the tree: longitudinal associations among American adolescents' civic engagement and family and school characteristics. Appl Dev Sci. 2023;28(3):302–322. doi: 10.1080/10888691.2023.2195183

[25] Aryabkina I, Spiridonova A, Belonogova L, et al. Sociocultural education as a key to fostering social responsibility in modern youth. Amazonia Investiga. 2024;13(78):113–123. doi: 10.34069/AI/2024.78.06.10

[26] Schaefer DR, Khuu TV, Rambaran JA, et al. How do youth choose activities? Assessing the relative importance of the microselection mechanisms behind adolescent extracurricular activity participation. Soc Networks. 2024;77:139–150. doi: 10.1016/j.socnet.2021.12.008

[27] Pong H-K, Leung CH. The impacts of community-service learning on career adaptability and on ethics and social responsibility of university students: an experimental study. J Educ Work. 2023;36(4):251–269. doi: 10.1080/13639080.2023.2174955

[28] Bandura A. The self system in reciprocal determinism. Am Psychol. 1978;33:344–358. doi: 10.1037/0003-066X.33.4.344

[29] Kim S, Jeong H, Cho H, et al. Extracurricular activities in medical education: an integrative literature review. BMC Med Educ. 2023;23(1):278. doi: 10.1186/s12909-023-04245-w 37087451 PMC10122317

[30] Ryan RM, Deci EL. Intrinsic and extrinsic motivation from a self-determination theory perspective: definitions, theory, practices, and future directions. Contemp Educ Psychol. 2020;61:101860. doi: 10.1016/j.cedpsych.2020.101860

[31] Ganotice FA Jr, Mendoza BN, Djiwandono T, et al. Students' motivation and engagement in interprofessional education: the mediating role of peer relatedness. Med Educ Online. 2024;29(1):2430593. doi: 10.1080/10872981.2024.2430593 39607950 PMC11610243

[32] Wu H, Li S, Zheng J, et al. Medical students' motivation and academic performance: the mediating roles of self-efficacy and learning engagement. Med Educ Online. 2020;25(1):1742964. doi: 10.1080/10872981.2020.1742964 32180537 PMC7144307

[33] King RB. Sociocultural and ecological perspectives on achievement motivation. Asian J Soc Psychol. 2021;25(3):433–448. doi: 10.1111/ajsp.12507

[34] Du W, Li Z, Xu Y, et al. The effect of parental autonomy support on grit: the mediating role of basic psychological needs and the moderating role of achievement motivation. Psychol Res Behav Manag. 2023;16:939–948. doi: 10.2147/PRBM.S401667 36992980 PMC10042245

[35] Bandura A. Social foundations of thought and action: a social cognitive theory. J Appl Psychol. 1986;12(1):169.

[36] Kifle BM. Characterizing disengagement in undergraduate education at MIT. Cambridge (MA): Massachusetts Institute of Technology; 2020.

[37] Ryan RM, Deci EL. Self-determination theory and the facilitation of intrinsic motivation, social development, and well-being. Am Psychol. 2000;55(1):68–78. doi: 10.1037/0003-066X.55.1.68 11392867

[38] Deci EL, Ryan RM. Self-determination theory: a macrotheory of human motivation, development, and health. Can Psychol. 2008;49(3):182–185. doi: 10.1037/a0012801

[39] Mulrooney H. Exploring participation in cocurricular activities among undergraduate students. New Dir Teach Phys Sci. 2017;1(12). doi: 10.29311/ndtps.v0i12.566

[40] Schüler J, Wolff W. What brings out the best and worst of people with a strong explicit achievement motive? The role of (lack of) achievement incentives for performance in an endurance task. Front Psychol. 2020;11:932. doi: 10.3389/fpsyg.2020.00932 32528364 PMC7264412

[41] Razack S, Risør T, Hodges B, et al. Beyond the cultural myth of medical meritocracy. Med Educ. 2020;54(1):46–53. doi: 10.1111/medu.13871 31464349

[42] Wei SG, Chen M, Zhang JC, et al. The theoretical basis, the questionnaire framework and the reliability and validity of the student survey of learning and development. Res High Educ Eng. 2015;(3):114–120.

[43] Chen M, Zhang JC, Wei SG, et al. The development and implementation of ‘Surveying the study and development of undergraduates’. Res High Educ Eng. 2015;(2):105–109.

[44] Hayes AF. Introduction to mediation, moderation, and conditional process analysis: a regression-based approach. 2nd. New York: Guilford Press; 2017.

[45] Podsakoff PM, MacKenzie SB, Lee JY, et al. Common method biases in behavioral research: a critical review of the literature and recommended remedies. J Appl Psychol. 2003;88(5):879–903. doi: 10.1037/0021-9010.88.5.879 14516251

[46] Alobaid SA, Beyari MB, Idris RB, et al. Students' perception of peer-students mentoring program “Big Sibling Mentoring Program” to complement faculty mentoring of first-year medical students in Saudi Arabia. Adv Med Educ Pract. 2024;15:837–843. doi: 10.2147/AMEP.S459942 39308481 PMC11414628

[47] Lee MH-Y, Iyengar Y, Budiansky D, et al. Exploring medical students' perceptions of peer-to-peer interactions related to applying to a surgical residency. J Surg Educ. 2024;81(2):193–201. doi: 10.1016/j.jsurg.2023.11.007 38142152

[48] Babuya J, Waruingi D, Mungujakisa D, et al. Medical students' knowledge, attitudes, and motivation toward antimicrobial resistance efforts in Eastern Uganda. PLoS One. 2025;20(2):e0314250. doi: 10.1371/journal.pone.0314250 39913348 PMC11801587

[49] Hatahet T, Alkhaledi A, Tello A, et al. Extracurricular activity based on undergraduates-postgraduates peer learning to promote student's academic research skills in developing countries higher education: experiences from Syria. Educ Res Eval. 2023;28(1-2):1–18. doi: 10.1080/13803611.2023.2259872

[50] Hoshina Y, Yada K, Maki H, et al. Medical English education in Japan: developing a curriculum to motivate students by providing visualization opportunities using near-peer teaching. J Med Invest. 2022;69(3.4):332–334. doi: 10.2152/jmi.69.332 36244792

[51] Achar Fujii RN, Kobayasi R, Claassen Enns S, et al. Medical students' participation in extracurricular activities: motivations, contributions, and barriers. A qualitative study. Adv Med Educ Pract. 2022;13:1133–1141. doi: 10.2147/AMEP.S359047 36176420 PMC9514135

[52] He L, Zhu C, Lei Y, et al. Humanistic value development through extracurricular activities in anatomical education. Int J Morphol. 2024;42(4):1119–1124. doi: 10.4067/S0717-95022024000401119

[53] Nshimiyimana A, Cartledge PT. Peer-teaching at the University of Rwanda - a qualitative study based on self-determination theory. BMC Med Educ. 2020;20(1):230. doi: 10.1186/s12909-020-02142-0 32689991 PMC7370529

[54] Da Silva Ezequiel O, Lucchetti ALG, Melo PF, et al. Factors associated with motivation in medical students: a 30-month longitudinal study. Med Sci Educ. 2022;32(6):1375–1385. doi: 10.1007/s40670-022-01651-5 36532391 PMC9755396

[55] Bin Abdulrahman KA, Alshehri AS, Alkhalifah KM, et al. The relationship between motivation and academic performance among medical students in Riyadh. Cureus. 2023;15(10):e46815. doi: 10.7759/cureus.46815 37954820 PMC10636236

[56] Swallow SR, Kuiper NA. Social comparison and negative self-evaluations: an application to depression. Clin Psychol Rev. 1988;8(1):55–76. doi: 10.1016/0272-7358(88)90049-9

[57] Alzaabi S, Nasaif M, Khamis A, et al. Medical students' perception and perceived value of peer learning in undergraduate clinical skill development and assessment: mixed methods study. JMIR Med Educ. 2021;7(3):e25875. doi: 10.2196/25875 34021539 PMC8317042

[58] Oliveira NM, Viana DA, Santos JR, et al. Engagement in extracurricular activities during medical school: a cross-sectional study on student motivations and challenges. J Med Educ Curric Dev. 2024;11:1–11. doi: 10.1177/23821205241296980 PMC11590139 39600962

[59] Findyartini A, Syah NA, Susilo AP, et al. Challenges and opportunities in cultivating medical students' competencies: participatory action research from a hierarchical cultural setting. Med Educ Online. 2023;28(1):2185122. doi: 10.1080/10872981.2023.2185122 36866628 PMC9987738

